# Unveiling candidate genes for metabolic resistance to malathion in *Aedes albopictus* through RNA sequencing-based transcriptome profiling

**DOI:** 10.1371/journal.pntd.0012243

**Published:** 2024-06-12

**Authors:** Xinyue Huang, Phillip E. Kaufman, Giridhar N. Athrey, Chris Fredregill, Michel A. Slotman

**Affiliations:** 1 Department of Entomology, Texas A&M University, College Station, Texas, United States of America; 2 Department of Poultry Science, Texas A&M University, College Station, Texas, United States of America; 3 Harris County Public Health, Mosquito & Vector Control Division, Houston, Texas, United States of America; University of California Davis School of Veterinary Medicine, UNITED STATES

## Abstract

*Aedes albopictus*, also known as the Asian tiger mosquito, is indigenous to the tropical forests of Southeast Asia. *Ae*. *albopictus* is expanding across the globe at alarming rates, raising concern over the transmission of mosquito-borne diseases, such as dengue, West Nile fever, yellow fever, and chikungunya fever. Since *Ae*. *albopictus* was reported in Houston (Harris County, Texas) in 1985, this species has rapidly expanded to at least 32 states across the United States. Public health efforts aimed at controlling *Ae*. *albopictus*, including surveillance and adulticide spraying operations, occur regularly in Harris County. Despite rotation of insecticides to mitigate the development of resistance, multiple mosquito species including *Culex quinquefasciatus* and *Aedes aegypti* in Harris County show organophosphate and pyrethroid resistance. *Aedes albopictus* shows relatively low resistance levels as compared to *Ae*. *aegypti*, but *kdr*-mutation and the expression of detoxification genes have been reported in *Ae*. *albopictus* populations elsewhere. To identify potential candidate detoxification genes contributing to metabolic resistance, we used RNA sequencing of field-collected malathion-resistant and malathion-susceptible, and laboratory-maintained susceptible colonies of *Ae*. *albopictus* by comparing the relative expression of transcripts from three major detoxification superfamilies involved in malathion resistance due to metabolic detoxification. Between these groups, we identified 12 candidate malathion resistance genes and among these, most genes correlated with metabolic detoxification of malathion, including four P450 and one alpha esterase. Our results reveal the metabolic detoxification and potential cuticular-based resistance mechanisms associated with malathion resistance in *Ae*. *albopictus* in Harris County, Texas.

## Author summary

The Asian tiger mosquito, *Aedes albopictus*, is an invasive species rapidly expanding worldwide. It is the main vector for several arboviruses, including dengue virus, Zika virus, and chikungunya virus. These viral diseases pose a substantial threat to global public health. *Ae*. *albopictus* has developed resistance to insecticides such as malathion, making its control more challenging. To uncover the genetic basis of this resistance, we conducted a study using RNA sequencing-based transcriptome profiling.

In this study, we obtained gene expression patterns in malathion-resistant and susceptible mosquitoes. The transcriptomic information allowed us to identify potential key genes in detoxification gene families associated with metabolic resistance to malathion. Our discovery provides insights into the molecular mechanisms behind malathion resistance in *Ae*. *albopictus*.

Our research not only contributes to the understanding of mosquito biology and control but also highlights the future direction for continued efforts in developing innovative strategies to mitigate rapid development of insecticide resistance in *Ae*. *albopictus*.

## Introduction

*Aedes albopictus* originates from tropical forests of Southeast Asia [1], later extending to India, and a few Pacific islands. However, *Ae*. *albopictus* can be found on all inhabited continents today [2]. The major reason for its introduction worldwide is the global transportation of dormant eggs inside used tires and other containers moved by air and sea transportation routes [3]. Given its limited natural dispersal ability, the rapid expansion of *Ae*. *albopictus* in North America can be ascribed to the human-aided transport of eggs and larvae in artificial containers [4]. Although *Ae*. *albopictus* was detected earlier, the first established population in the continental United States was recorded in Houston (Harris County), Texas in 1985 [5]. By 2014 *Ae*. *albopictus* had spread to at least 32 states in the United States [6]. Mosquitoes belonging to the genus *Aedes* are a significant threat to human health owing to transmission of viruses [7]. *Aedes albopictus* is known as a vector of several viruses, including Zika [8], dengue [2], chikungunya [9], and a potential vector of yellow fever [10]. Due to the lack of specific vaccines and effective treatments for many mosquito-borne viral diseases, prevention and control of these diseases primarily rely on vector control, with extensive application of insecticides [11]. Therefore, the development of insecticide resistance in arthropod vectors, including *Aedes* mosquitoes, has become a serious public health concern [12].

Compared to another dengue vector, *Ae*. *aegypti*, reports of insecticide resistance in *Ae*. *albopictus* are fewer [13]. Although more limited, insecticide resistance has been reported in *Ae*. *albopictus* in many areas of the world, with most originating in Asia and the Americas [14]. For example, resistance to organophosphates have been reported in *Ae*. *albopictus* populations in Malaysia [15], Singapore [16], and China [17]. In the United States, malathion resistance has been reported in *Ae*. *albopictus* populations from Texas [18], Illinois [19], and Florida [20]. Liu et al. [21] reported a low level of tolerance to malathion in *Ae*. *albopictus* sampled from Alabama and Florida. Similarly, resistance against dichlorodiphenyltrichloroethane (DDT) and malathion was reported in *Ae*. *albopictus* populations from Florida and New Jersey [14,22]. These findings suggest a capacity to express cross-resistance among insecticide classes and indicates the importance of insecticide resistance monitoring in *Ae*. *albopictus*.

Two major mechanisms of insecticide resistance are widespread in mosquitoes, including metabolic detoxification and target site insensitivity. Although less knowledge is available about insecticide resistance mechanisms in *Ae*. *albopictus* compared to *Ae*. *aegypti*, the knockdown resistance (*kdr*) mutation has been reported in *Ae*. *albopictus* worldwide [14]. The primary mechanism of malathion and other organophosphates (OPs) is through inhibition of acetylcholinesterase (AchE) [23]. Mutations on AChE such as G119S, F290V and F331W have been previously reported to associate with resistance to OPs in *Anopheles gambiae* and *Culex pipiens and Cx*. *tritaeniorhynchus* [24–29]. However, *kdr* mutations reported in *Ae*. *albopictus* are predominantly mutations in the voltage sensitive sodium channel (VSSC) or voltage-gated sodium channel (VGSC) encoded by the *Vssc* gene [14]. One study investigating two *Ae*. *albopictus* populations from China aimed to identify modification on AchE *ace-1* gene, but this study failed to detect any mutations [30]. Overexpression of cytochrome P450s, glutathione S-transferases (GSTs), and esterases contribute to metabolic resistance in *Ae*. *albopictus* [22,31,32], while overexpression of the P450 gene, *Cyp6p12*, confers pyrethroid resistance in *kdr*-free *Ae*. *albopictus* [31]. Overexpression of detoxification genes can be triggered through diverse mechanisms, such as regulation by transcription factors [33] and copy number variation (CNV) [34]. CNV is a major resource of evolutionary novelties, as well as an important source of short-term adaptive responses to resist stress, such as occurs with insecticide selection [35]. Particularly, CNV in the *CCEae3a* and *CCEae6a* genes, which confers malathion resistance in *Ae*. *albopictus* in Athens, Greece and Florida, USA [34].

Transcriptome profiling and differential gene expression (DGE) analysis between resistant and susceptible *Ae*. *albopictus* has been employed for revealing relationship between amplified carboxylesterase genes and temephos resistance [36]. Xu et al. combined transcriptome profiling and RNA interference (RNAi) to identify and validate differentially expressed genes associated with pyrethroid resistance in *Ae*. *albopictus* [37]. Transcriptome profiling, DGE analysis and RNAi techniques were employed in our previous research on *Cx*. *quinquefasciatus* to reveal the involvement of P450 genes, *Cyp325bc1* and *Cyp9m12*, in malathion resistance [38]. These studies support the feasibility and reliability of using transcriptome to detect candidate genes in the metabolic detoxification process in *Ae*. *albopictus*.

In this study, we analyzed the transcriptome profiles of *Ae*. *albopictus* mosquitoes collected from Harris County and from a malathion susceptible laboratory colony. We focused on identifying differential gene expression patterns, particularly in genes associated with three major detoxification superfamilies: P450s, GSTs, and esterases. We present findings on the differences in gene expression between malathion-resistant and malathion-susceptible *Ae*. *albopictus*.

## Materials and methods

The Mosquito and Vector Control Division of Harris County Public Health (HCPH) conducted mosquito collections and Centers for Disease Control and Prevention (CDC) bottle assays. Four experimental groups were established in this study, Wild group (WI), Colony group (CO), Malathion Resistant group (MR) and Malathion Susceptible group (MS). The field-captured (WI) group *Ae*. *albopictus* originated as eggs collected at operational area 51 in Houston, Texas (S1 Fig). At least 30 ovicups were placed at 15–20 sites within operational area 51 based on vegetation coverage and minimal human disturbance with up to three ovicups placed per site. Ovicups were placed 1.5–3 m apart from each other. Collected eggs were raised to adulthood in the HCPH insectary under controlled conditions at 26±1°C and 75 ± 5% relative humidity (RH), under a 12:12 hour light:dark (L:D) photoperiod. The sampling and selecting for WI, MR and MS group were processed the same way as described in Huang et al [38]. Briefly, WI group *Ae*. *albopictus* eggs were collected, and a portion of the eggs were reared to the adult stage. Reared 3-day-old females were exposed in groups of 15–30 as to the diagnostic concentration of malathion (400 μg/bottle) for 60 minutes, based on instructions from the CDC CONUS (Continental US) manual for bottle assay [39]. Mosquitoes were considered dead when unresponsive to manual stimulation. Following a 30-minute exposure (CDC diagnostic designation), knocked-down mosquitoes were immediately collected and transferred into RNAlater-ICE solution. These mosquitoes knocked-down in the first 30 minutes during the bottle assay were classified as Malathion Susceptible (MS) group [39]. Mosquitoes classified as live after a one-hour bottle assay exposure were transferred to a clean container and held for 24 hours under standard insectary conditions. After the 24-hour holding period, all live mosquitoes were labeled as Malathion Resistant (MR) group [39] and placed into RNAlater-ICE solution. The laboratory-maintained susceptible ATM-NJ95 colony strain (CO) originated from Keyport, NJ, USA in 1995 [22]. Eggs of this colony were obtained from the BEI Resources (www.beiresources.org) and were reared without exposure to insecticide under the same conditions as described above. Mortality curves for CDC bottle assay were generated using ggplot2 package [40] within R version 4.1.1 (https://www.r-project.org).

To identify gene expression patterns involved in *Ae*. *albopictus* metabolic resistance against malathion, we used an RNA sequencing (RNA-Seq) approach. Before RNA extraction from mosquitoes, we first performed morphological identification on each mosquito based on the presence of the bold black shiny scales and distinct silver-white scales on the palpus and tarsi [41]. Four experimental groups were included in total and each experimental group contained five technical replicates. Ten mosquito individuals were pooled as one biological replicate and RNA extraction was processed as described in Huang et al [26]. Quality control of RNA samples was performed with the Agilent 2100 Bioanalyzer at the Texas A&M University Genomics Facility (TxGen). Samples showing evidence of RNA degradation were excluded. Qualified RNA samples were sequenced at the TxGen with the same equipment and settings as described in Huang et al [26].

The Genome Analysis Toolkit (GATK) Best Practices was optimized to process RNA sequencing data [42] for transcriptome analysis. We first performed quality control steps on the raw sequence data starting with FASTQ files. After the adaptor trimming and quality control step was completed with TrimGalore version 0.6.4_dev [43] using a Phred score threshold of 20 and a minimum sequence length threshold of 20 bp, trimmed reads were mapped to the whole genome sequences of *Ae*. *albopictus* laboratory Foshan strain using STAR version 2.7.3a [44]. *Aedes albopictus* genome data were downloaded from VectorBase [45] and its structural annotation version was AaloF1.2. Two-pass alignment was applied for high sensitivity and accuracy following the index building for the reference genome. Picard tools version 2.20.1 (http://broadinstitute.github.io/picard) was used to identify and add Read Group (RG) for unsorted BAM files obtained from the previous step. The BAM files belonging to the same technical replicate in each experimental group that were sequenced on different lanes were merged. In the end, reads assigned to genomic features were counted using featureCounts version 1.6.0 [46].

Differential gene expression analysis was performed using edgeR version 3.36.0 [47] within R version 4.1.1 (https://www.r-project.org). After filtering out low-count data (genes with fewer than two samples with counts-per-million values greater than 1), a Trimmed Mean of M-values (TMM) method [48] was used to compute normalization factors in edgeR. We used the R package SsizeRNA [49] to check the power of our experimental design for RNA-seq based on the dispersion estimate obtained from RNA-seq data, the number of replicates, and the average depth per gene obtained from the uniquely aligned reads. We used the “check.power” function in the package to perform this test. We ran 50 sets of simulations of the power analysis to estimate the Benjamini-Hochberg Average estimated power [49]. We proceeded with the interpretation of the data based on the outcome of this power test. The pairwise comparisons between every two experimental groups was performed with the likelihood ratio test (LRT) method. We employed ClustVis [50] to produce a cluster heatmap, facilitating a direct visualization of the expression information of differentially expressed detoxification genes (DEGs) belonging to P450, GST and esterase superfamilies across the four experimental groups.

Gene ontology enrichment analysis was performed with gene lists generated from the DGE analysis step using a graphical tool, ShinyGO version 0.76 [51]. ShinyGO supports enrichment analysis based on annotation databases acquired from Ensembl [52] and STRING-db [53], which enable functional interpretation of gene lists through integrating them into known molecular pathways. DEGs with a false discovery rate (FDR for DEG) cutoff value of less than 0.05 detected in the DGE analysis step were imported for functional classification. After that, fold enrichment (FE) values were calculated with a false discovery rate (FDR for FE) from the percentage of DEGs belonging to a specific pathway as described in Huang et al [26].

## Results

**Fig 1:**
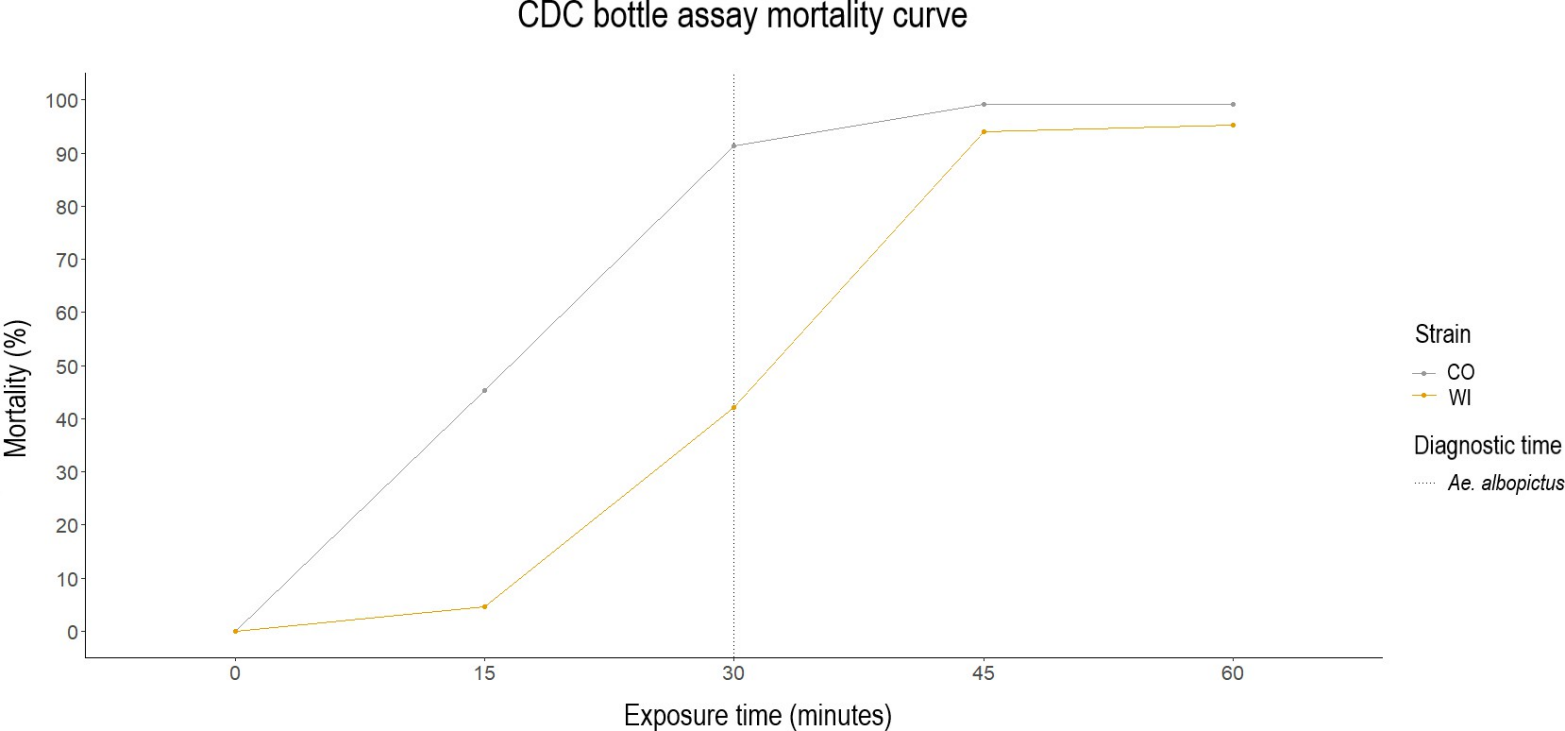
CDC bottle bioassay mortality curves were generated with ggplot2 package within R for 60-minute exposure to malathion on a field-captured (WI) strain and a laboratory-maintained susceptible (CO) strain. A dose of 400 μg malathion/bottle was used. Mortality curves of WI and CO are represented by yellow and gray, respectively. The straight dotted line represents the diagnostic time to malathion for *Ae*. *albopictus* (30 minutes) based on the manual of CDC bottle bioassay.

The CDC bottle bioassays were performed on a wild-collected (WI) strain and a laboratory-maintained susceptible ATM-NJ95 (CO) strain (Fig 1). Malathion resistant (MR) and malathion susceptible (MS) groups were divided from WI group based on the CDC bottle assay results. Mortality of the CO group at the diagnostic time was 91.25% with a 95% confidence interval from 79.23% to 100%. Mortality of the WI group at the diagnostic time was 42.05% with a 95% confidence interval from 32.75% to 51.35%. Mortality in control bottles for both CO and WI group was 0. Therefore, no correction for mortality was needed in this study [39].

cDNA libraries were constructed and sequenced for four experimental groups. Each experimental group was represented by five replicates. A total of more than 9.56 billion 100-bp reads were generated, including total reads ranging from 2,087,899,176 in the MR group to 2,570,347,932 in the MS group (Table 1). After the filtering step was performed with TrimGalore, we successfully mapped more than 75% of reads to the *Ae*. *albopictus* Foshan genome (version: AaloF1.2) [54], including mapping reads ranging from 75.33% in the MS group to 77.94% in the CO group. Filtered reads were assembled into 18,294 genes in the *Aedes albopictus* Foshan genome (version: AaloF1.2). Our power analysis indicated that our experiment had 81.60% power to detect significant differences at an estimated effect size of log_2_ fold change (Log FC) of 2 or greater, and an FDR value of <0.05. As 80% power is considered the standard threshold for power [55], our experiment meets or exceeds this traditionally accepted standard for power, depending on a minimum defined effect size. For genes that showed greater than Log FC >3 differences, we had 99.88% power to detect true differences.

**Table 1:** Summary statistics of sequencing data for *Aedes albopictus* transcriptome analysis including mapping totals, Q20 and GC percentage.

Experimental Group	Read Length (Filtered)	Number of Reads (Filtered)	Total Reads Mapped (%)	Q20 (%)^1^	GC (%)^2^
Wild	99.36	2,426,027,422	1,879,781,574 (77.48)	99.36	46.32
Colony	99.13	2,414,446,550	1,881,839,002 (77.94)	99.13	45.81
Malathion Resistant	98.45	2,077,814,128	1,608,357,334 (77.41)	98.45	46.48
Malathion Susceptible	99.31	2,562,725,004	1,930,627,636 (75.33)	99.31	47.42

^1^Q20% = percentage of bases with Phred quality score (Q score) higher than 20.

^2^GC% = percentage of G + C in the reads.

We identified 140 genes coding for detoxification enzymes from the P450, GST and esterase superfamilies. Furthermore, we discovered 75 differentially expressed genes (DEGs) with a false discovery rate (FDR) of less than 0.05 from these three detoxification gene families. We identified 3,921 up-regulated genes and 3,628 down-regulated genes in the WI group as compared to the CO group. We found 71 DEGs in the comparison between WI and CO groups, including 50 P450 genes, 19 GST genes, and two esterase genes (S1 Table). We identified 386 up-regulated genes and 74 down-regulated genes in the MR group as compared to the MS group. Four DEGs (FDR < 0.05) were found in the comparison between the MR and MS groups, including one P450 gene, two GST genes, and one esterase gene. In addition, we identified 22 DEGs with a *p*-value < 0.05 between the MR and MS groups, including 12 P450 genes, eight GST genes, and two esterase genes (S2 Table). Altogether, we identified 12 detoxification genes of interest (Table 2). Transcripts Per Million (TPM) of genes from the three major detoxification families ranged from 0.04 to 10206.06 and had an average value of 171.19 (S3 Table). Significant up-regulation of genes such as *Cyp6z18*, *Cyp6m17*, *Cyp6d4*, and carboxy/choline esterase (CCE) alpha esterase were detected in both transcriptome comparisons. We also observed significant down-regulation of the cytochrome P450 *304a1* gene in both transcriptome comparisons.

**Table 2:** Summary for differences in gene expression (DGE) involved in malathion resistance in *Aedes albopictus* (FDR <0.05 in both comparisons between the WI and CO groups and between the MR and MS groups).

AALF annotation^1^	Gene name	Gene location (Sense/Antisense)^2^	Log FC^3^ (WI versus CO)^4^	Log FC^3^ (MR versus MS)^4^	Log CPM^5^
AALF021146	glutathione transferase	JXUM01S005103: 47,668–58,409(+)	1.06	-1.84	6.06
AALF007799	Carboxy/choline esterase Alpha Esterase	JXUM01S000186: 122,194–130,405(-)	0.63	0.59	5.39
AALF015905	glutathione transferase	JXUM01S003629: 146,097–147,373(-)	0.77	-1.64	4.28
AALF015440	cytochrome P450 *Cyp6z18*	JXUM01S003515: 237,786–239,324(-)	0.77	0.68	6.11
AALF027556	cytochrome P450	JXUM01S000943: 237,491–256,802(+)	0.71	-0.64	4.57
AALF018946	cytochrome P450 *Cyp6m17*	JXUM01S000525: 244,188–245,733(-)	0.65	0.50	4.62
AALF007720	cytochrome P450 *6d4*	JXUM01S013578: 11,732–12,808(+)	1.36	3.40	0.49
AALF004240	cytochrome P450	JXUM01S001348: 75,217–77,371(+)	0.99	-1.05	5.30
AALF006638	cytochrome P450 *304a1*	JXUM01S001613: 42,481–63,255(-)	-0.83	-1.89	2.18
AALF009469	cytochrome P450	JXUM01S000226: 100,365–105,171(+)	0.48	-1.39	7.49
AALF021145	glutathione transferase	JXUM01S005103: 32,407–46,936(+)	0.51	-1.79	6.76
AALF020660	cytochrome P450	JXUM01S004952: 54,414–78,681(+)	0.51	-0.93	9.62

^1^AALF = *Aedes albopictus* Foshan

^2^Gene location, as described in VectorBase, (S/NS) = Sense = “+” and Non-sense = “-” strand

^3^Log FC = log_2_ fold change calculated by edgeR, positive values represent up-regulated gene expression and negative values represent down-regulated gene expression

^4^WI, CO, MR, MS = mosquito strain sub-types representing wild, colony, malathion resistant and malathion susceptible

^5^Log CPM = log_2_ average counts-per-million calculated by edgeR

**Fig 2:**
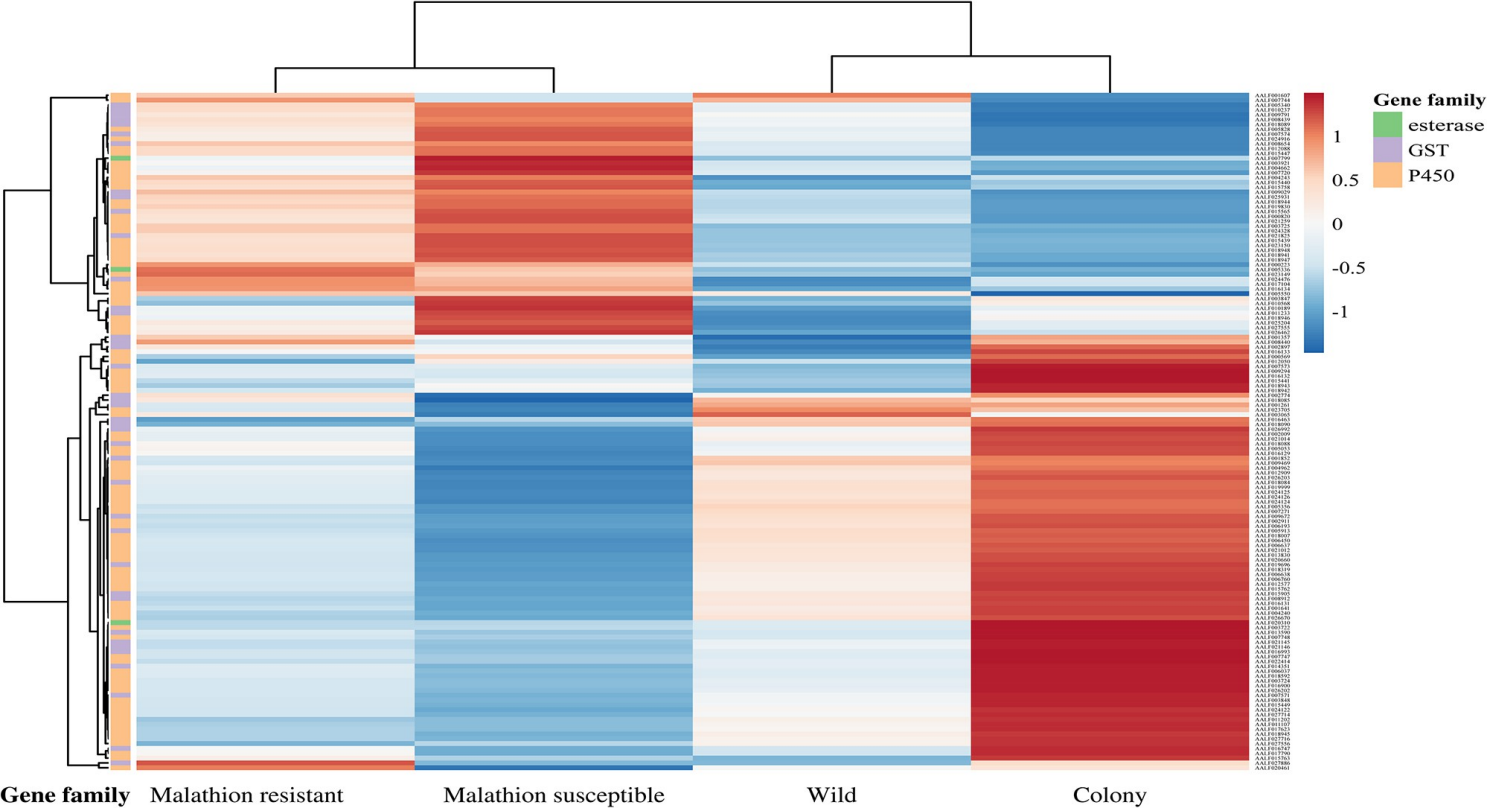
The cluster heatmap was generated by ClustVis to illustrate gene expression data. The heatmap was generated with 140 genes coding for detoxification enzymes from the P450, GST and esterase superfamilies from gene expression profiling. Each row in the grid represents a gene, and each column in the grid represents an experimental group. Gene expression levels are color coded, with down-regulation as blue and up-regulation as red. The intensity of the color represents the relative expression level normalized using unit variance scaling. Genes from esterase, GST and P450 family are represented by green, purple, and orange, respectively.

The cluster heatmap visualizes the expression profile for 140 genes from the three major detoxification superfamilies, including the P450, GST and esterase gene families (Fig 2). We observed a similar pattern of expression within bioassay-selected groups (MR and MS) and uninduced groups (WI and CO), respectively.

**Fig 3:**
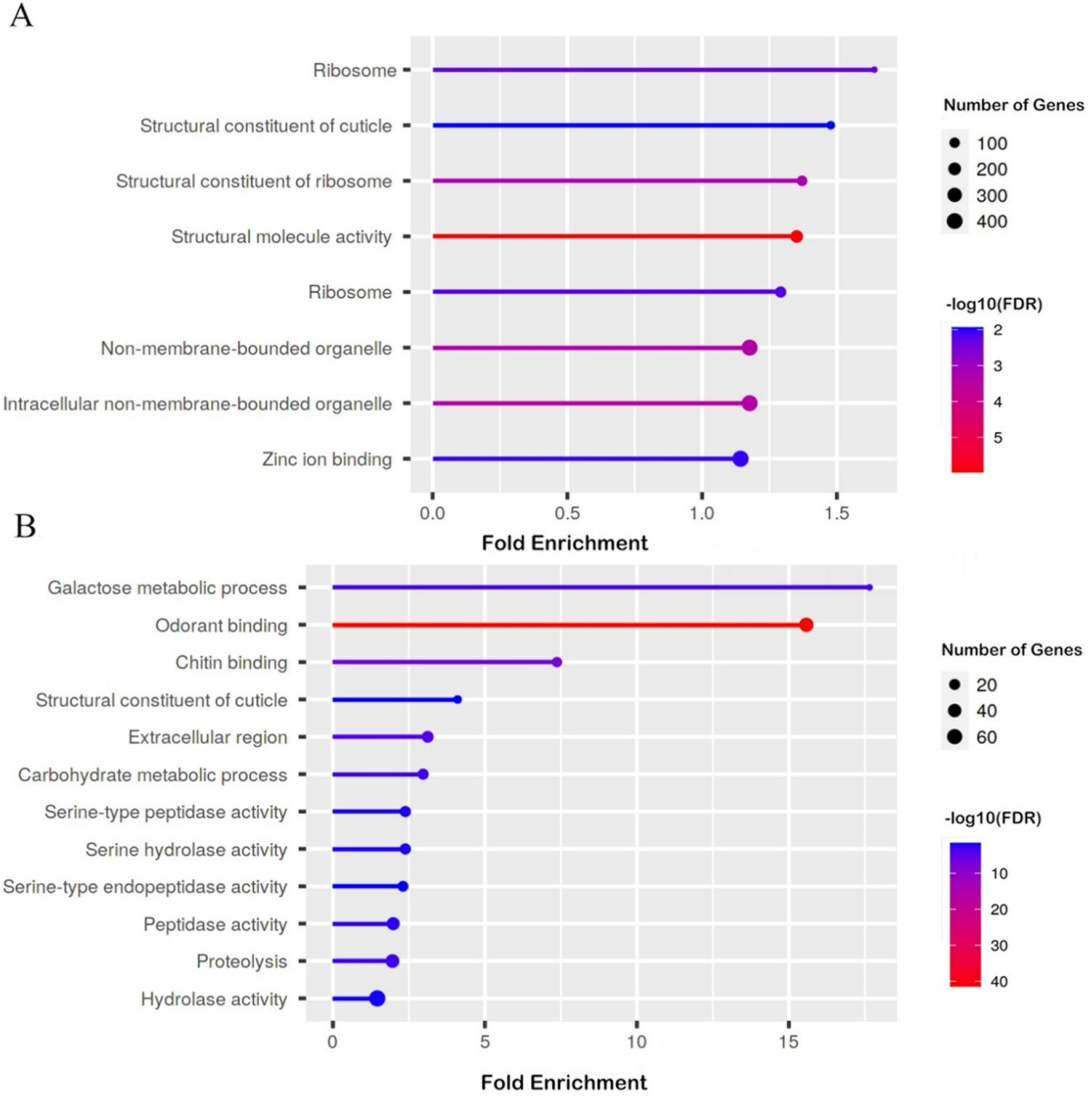
The ShinyGO Pathway Analysis illustrates the significant pathways comprising differentially expressed genes (DEGs) with a significant level (FDR value for DEG less than 0.05) when these genes are mapped to the molecular pathways and functional categories in the available *Aedes albopictus* databases. The size of a circle represents the DEG number included in one specific pathway. Fold Enrichment (FE) was calculated from dividing the percentage of DEGs belonging to a pathway by the corresponding percentage of genes in the background. FDR for FE represents the probability that the enrichment occurs randomly. (A) The eight significant pathways with the most DEGs (FDR for DEG with a cutoff value of less than 0.05) in the comparison between *Ae*. *albopictus* WI and CO groups were labeled in the chart. (B) The 12 significant pathways with the most DEGs in the comparison between *Ae*. *albopictus* MR and MS groups were labeled in the chart.

Pathway analysis illustrates the significant pathways containing DEGs detected in the previous transcriptome comparison steps. DEGs between WI group and CO group were classified into eight pathways (S4 Table). These eight significant pathways are presented (Fig 3A). DEGs between MR group and MS group were classified into 15 pathways (S5 Table). Transcriptome comparisons between the MR and MS groups show the 12 significant DEG pathways using the FE approach (Fig 3B).

The structural constituent of the cuticle was one of the top pathways in the transcriptome comparison between the WI and CO groups, with an FDR value for FE of 5.90e-03. It was also one of the top pathways in the transcriptome comparison between the MR and MS groups, with the FDR value for FE of 0.043. However, well annotated cuticle-related DEGs with significant FC patterns were not observed in either comparison.

## Discussion

In this study, we identified candidate genes for involvement in malathion resistance through transcriptome profiling of resistant and susceptible *Ae*. *albopictus*. We performed a transcriptome comparison between a field-collected (WI) group and a laboratory-maintained susceptible (CO) group to study constitutive overexpression of key genes. We identified 7,549 DEGs between the WI and the CO groups, including 193 DEGs from three major detoxification gene families. Our result indicates that the expression level of many genes, including but not limited to detoxification genes, differs considerably between the field-collected mosquitoes and laboratory-maintained unexposed *Ae*. *albopictus*. Chen et al. have suggested that the large genome repertory and plasticity of *Ae*. *albopictus* may be contributing factors to its successful worldwide expansion [54]. Our findings support the high adaptive potentiality of *Ae*. *albopictus* gene expression plasticity under the impacts of human activities in urban areas, such as the application of insecticides.

Furthermore, we explored overexpression of detoxification genes induced by malathion through transcriptome profile comparison between malathion-resistant (MR) and susceptible (MS) *Ae*. *albopictus*. We identified 460 DEGs between the MR and the MS groups, including 32 DEGs from three major detoxification gene families. Overexpression of three P450 genes, *Cyp6z18*, *Cyp6m17*, *Cyp6d4*, and one CCE alpha esterase was observed in both the MR and the WI groups in this study. Prior research in Tanzania showed that *Cyp6z18* expression in resistant *Cx*. *quinquefasciatus* is increased 8-fold [56]. This result has been further validated by increased mortality of bendiocarb and deltamethrin-resistant mosquitoes in synergist assays using the P450 inhibitor piperonyl butoxide [56]. Interestingly, an organophosphate larvicide, temephos, has been reported to inhibit the expression of *Cyp6z18* in larval stage *Ae*. *albopictus* [57]. These findings might be explained by different expression profiles between developmental stages or between species. Chan et al. also demonstrated that CuSO_4_ can induce the significant overexpression of *Cyp6m17* [57]. Preexposure to CuSO_4_ induces the activity of multiple P450s and contributes to tolerance towards permethrin and temephos in *Ae*. *aegypti* larvae [58]. Our work and previous studies indicate that cross-exposure of environmental xenobiotics, such as malathion or CuSO_4_, may trigger metabolic detoxification mechanisms in mosquitoes. One of the candidate genes we identified, *Cyp6d4* was also more highly expressed in pyrethroid resistant *Ae*. *albopictus* from southern China[37]. In *Drosophila melanogaster Cyp6d4* expression is induced by stress-responsive xenobiotic treatments, such as paraquat and tunicamycin [59]. However, a *Cyp6d4* null mutant in *D*. *melanogaster* showed no significant alteration in insecticide resistance. The expression of *Cyp304a1* was significantly down-regulated in our study in both the MR and the WI groups. To find down-regulated of *Cyp304a1* in both groups is interesting, as up-regulation of *Cyp304a1* was suggested to contribute to tolerance of insecticide in *Bactrocera dorsalis* adults after exposed to high-sucrose diets during their larval stage [60]. An indication of its function is provided by a recent study on *Ae*. *albopictus* larvae suggesting that it is induced by haedoxan A and participates in haedoxan A detoxification [61]. Further studies are needed to elucidate mechanisms of gene regulation for the cytochrome P450 system. For instance, remarkable differential expression of P450 genes might directly contribute to insecticide resistance or may occur corresponding to other physiological responses against insecticides.

The alpha esterase of the carboxylesterase family (VectorBase ID: AALF007799) that is significantly up-regulated in both the MR and WI groups is orthologous to the alpha esterase gene *CCEae6a* (VectorBase ID: AAEL005122) from *Ae*. *aegypti*. The expression of this transcript, had a 6.1-fold overexpression in a temephos-selected resistant *Ae*. *albopictus* strain compared to a susceptible control strain [62]. Temephos in their research and malathion used in our experiment are organophosphate insecticides. Quantitative amplification in esterases has been widely documented in organophosphate-resistant strains of mosquitoes, such as *Cx*. *pipiens* [63] and *Cx*. *quinquefasciatus* [64]. Particularly, elevated alpha esterase activities have been recorded with permethrin and organophosphate tolerance in many mosquitoes, including *Ae*. *aegypti* [65] and *Ae*. *albopictus* [66]. Overall, these results indicate that esterases may participate in metabolic detoxification of organophosphate insecticides in *Ae*. *albopictus*, though we have little understanding of the underlying mechanism.

We observed highly differentiated patterns of expression between bioassay-selected groups (MR and MS) and uninduced groups (WI and CO) in the P450, GST and esterase gene families (Fig 2). The overexpression of similar detoxification genes in the MR and MS groups after exposure to malathion indicated a xenobiotic-induced mode in regulation of detoxification genes in *Ae*. *albopictus*. Some detoxification genes were specifically up-regulated in the MR group, while these were down-regulated in the MS group. Of note, the expression level of most detoxification genes in the MS group were higher than comparable genes in the MR group. This suggests that a small number of genes might determine the malathion resistance in *Ae*. *albopictus* based on samples from Harris County.

Additionally, expression patterns in the WI group were similar to the CO group, indicating a constitutive mode in detoxification gene expression. However, the expression level of detoxification genes in the WI group was neither highly up-regulated nor greatly down-regulated compared to the CO group. A possible explanation for this result is the increase in fitness cost caused by detoxification gene overexpression. Fitness costs incurred by insecticide resistance have been widely suggested in previous studies as overexpression of genes associated in resistance requires reallocation of energy and other resources at the expense of other metabolic processes essential for adaption and survival [67]. The difference in the expression profile of the CO group as compared to the other three groups collected in Harris County might be related to environmental variables, such as insecticide treatments over Harris County after Hurricane Harvey [68] and exposure to stress like temperature or toxin of *Bacillus thuringiensis israelensis* [69].

In addition to target-site mutation and metabolic detoxification, behavioral adaptation and cuticular modification are also important adaptive strategies that avoid contact with or prevent penetration of insecticides [70,71]. Ontology analysis revealed the cellular components, biological processes and molecular functions determining differential gene expression among the four experimental groups. We reported eight significantly enriched pathways involving DEGs from transcriptome comparison between WI and CO groups, and 12 significantly enriched pathways involving DEGs from the transcriptome comparison of the MR and MS groups (Fig 3). Interestingly, the structural constituent of cuticle (Term GO: 0042302) was one of the significantly enriched pathways in both transcriptome comparisons. In addition, chitin binding (Term GO: 0008061) was significantly enriched with an FDR value of 1.03e-07 in a comparison between the MR and MS groups. Chitin is an important biopolymer constituting the exo- and endocuticles in insects [72]. Cuticle thickening has been suggested to correlate with resistance through reducing penetration amount or absorption rates of insecticide in mosquitoes, such as *Anopheles funestus* [73], *An*. *gambiae* [74] and *Ae*. *aegypti* [75]. Consistent overexpression of cuticular protein genes has been reported in comparison between permethrin-resistant and unexposed *Ae*. *albopictus*, indicating reduced penetration caused by cuticle thickening as an important mechanism of pyrethroid resistance [31]. Our discoveries in DEG and ontology analysis suggest that alteration of the cuticle might contribute to malathion resistance in *Ae*. *albopictus*.

Pathway analysis reduces the complexity of extracting meanings from thousands of differentially expressed genes by grouping them into hundreds of pathways [76]. However, accuracy in the interpretation of pathway analysis is determined by pathway analyzing methods and completeness of annotations. Pathway analysis using ShinyGO herein can be classified as one over-representation analysis (ORA) approach, characterized by inputting DEGs, counting the proportion of DEGs in genes of one specific pathway, and repeating this process for background genes and eventually testing every pathway for over- or underrepresentation in input DEGs. A frequent limitation of ORA approaches is information loss caused by ignoring non-significant genes (e.g., *p*-value < 0.05 but FDR ≥ 0.05) that may be related to statistical power and effect sizes. It should be also noted that the *Ae*. *albopictus* genome is highly repetitive and this brings extra challenges for sequencing and assembly [77]. Available *Ae*. *albopictus* reference genome, such as AaloF1.2 used in this study [54], can be further developed to provide a higher confidence for structural and functional annotation [78].

We did not screen for modification on AchE *ace-1* gene in this study. Knockdown resistance to malathion and other OPs due to genetic modification of AchE have been widely reported in other mosquito species, such as *An*. *gambiae*, *Cx*. *pipiens* and *Cx*. *tritaeniorhynchus* [24–29]. The potential contribution of AchE modification to malathion resistance merits thorough investigation and elucidation in future studies.

We used whole mosquito instead of specific body parts to obtain a comprehensive view of gene expression profiles and identify key genes associated with malathion resistance in *Ae*. *albopictus*. It is generally considered that the midgut and fat body tissue are primary detoxification organs where most insect detoxification genes such as P450s are expressed [79]. For example, Liu et al. demonstrated that the P450 gene *Cyp6aa7* showed reduced expression in head, elevated expression in thorax, and attained its peak expression level in the abdomen tissue in permethrin-susceptible and resistant strains of *Cx*. *quinquefasciatus* [80]. But tissue-specific expression of detoxification genes in other organs, such as brain, may also be important for fitness cost and response to insecticide resistance [81]. Tissue-specific transcriptome analyses at different developmental stages of *Ae*. *albopictus* are needed to expand our knowledge of the regulatory mechanisms, and the temporal and spatial distribution of detoxification genes.

## Conclusions

Our results indicate that metabolic detoxification mechanisms may participate in malathion resistance in *Ae*. *albopictus* in Harris County. We identified 12 specific detoxification genes as candidates for the metabolization of malathion, although further studies are required to confirm the function and regulatory mechanism of these genes. P450 gene *Cyp6z18* and *Cyp6d4* as well as the alpha esterase gene *CCEae6a* have been reported to associate with insecticide resistance while P450 gene *Cyp6m17* is first identified. Our study also suggests that cuticular thickening might be one of the important mechanisms in malathion resistance in *Ae*. *albopictus*. This work expanded current knowledge about metabolic detoxification in an important vector, *Ae*. *albopictus* particularly under exposure to malathion and provided a constructive reference for further exploration of detoxification gene regulation mechanisms.

## Supporting information

S1 Fig Map of Harris County Public Health operational areas. There are 268 operational areas divided by HCPH in Houston. The field-captured Aedes albopictus originated as eggs collected from operational area 51. Area 51 is filled with red color. This map shows the Harris County boundary, presented as a map image layer created using PHES_AGO on 7 June 2017, and updated on 7 May 2020. This map also shows the operational area boundaries, presented as a map image layer crafted by PHES_AGO on 4 November 2016, and updated on 9 May 2020. The map layer for county boundary (Map service: Harris County boundary masked) (https://www.arcgis.com/home/item.html?id=a8aa2ef4067348c79ccea62857a2f623) and the layer for operational area boundaries in Harris County (MVCD_Operational_Areas) (https://www.arcgis.com/home/item.html?id=66643535e01b42d3aae5d4647f5e1a6c) were generated using ArcGIS (https://www.arcgis.com/home/webmap/viewer.html; ESRI, CA) by HCPH and are publicly available. There are no special restrictions or limitations on the terms of use of the layers integrated into this map. This map was completed by assembling these two layers and by coloring the research areas using the ArcMap 10.8 software (ESRI, CA).
(PDF)

S1 Table Differentially expressed genes between WI and CO groups. (XLSX)

S2 Table Differentially expressed genes between MR and MS groups. (XLSX)

S3 Table TPM value of genes from the three major detoxification families. (XLSX)

S4 Table Significant pathways between WI and CO groups. (XLSX)

S5 Table Significant pathways between MR and MS groups. (XLSX)
