# Supplementary material for: Unveiling candidate genes for metabolic resistance to malathion in Aedes albopictus through RNA sequencing-based transcriptome profiling
Source: PLoS Negl Trop Dis. 2024 Jun 12;18(6):e0012243. doi: 10.1371/journal.pntd.0012243 (PMC11168629; doi:10.1371/journal.pntd.0012243)
Supplement: S1 Fig — Area 51 is filled with red color. This map shows the Harris County boundary, presented as a map image layer created using PHES_AGO on 7 June 2017, and updated on 7 May 2020. This map also shows the operational area boundaries, presented as a map image layer crafted by PHES_AGO on 4 November 2016, and updated on 9 May 2020. The map layer for county boundary (Map service: Harris County boundary masked) (https://www.arcgis.com/home/item.html?id=a8aa2ef4067348c79ccea62857a2f623) and the layer for operational area boundaries in Harris County (MVCD_Operational_Areas) (https://www.arcgis.com/home/item.html?id=66643535e01b42d3aae5d4647f5e1a6c) were generated using ArcGIS (https://www.arcgis.com/home/webmap/viewer.html; ESRI, CA) by HCPH and are publicly available. There are no special restrictions or limitations on the terms of use of the layers integrated into this map. This map was completed by assembling these two layers and by coloring the research areas using the ArcMap 10.8 software (ESRI, CA). (PDF) [file pntd.0012243.s001.pdf]

S1 Fig. Map of Harris County Public Health operational areas. There are 268 operational areas divided by HCPH in Houston**.** The field-captured *Aedes albopictus* originated as eggs collected from operational area 51. Area 51 is filled with red color. This map shows the Harris County boundary, presented as a map image layer created using PHES_AGO on 7 June 2017, and updated on 7 May 2020. This map also shows the operational area boundaries, presented as a map image layer crafted by PHES_AGO on 4 November 2016, and updated on 9 May 2020. The map layer for county boundary (Map service: Harris County boundary masked) (https://www.arcgis.com/home/item.html?id=a8aa2ef4067348c79ccea62857a2f623) and the layer for operational area boundaries in Harris County (MVCD_Operational_Areas) (https://www.arcgis.com/home/item.html?id=66643535e01b42d3aae5d4647f5e1a6c) were generated using ArcGIS (https://www.arcgis.com/home/webmap/viewer.html; ESRI, CA) by HCPH and are publicly available. There are no special restrictions or limitations on the terms of use of the layers integrated into this map. This map was completed by assembling these two layers and by coloring the research areas using the ArcMap 10.8 software (ESRI, CA).

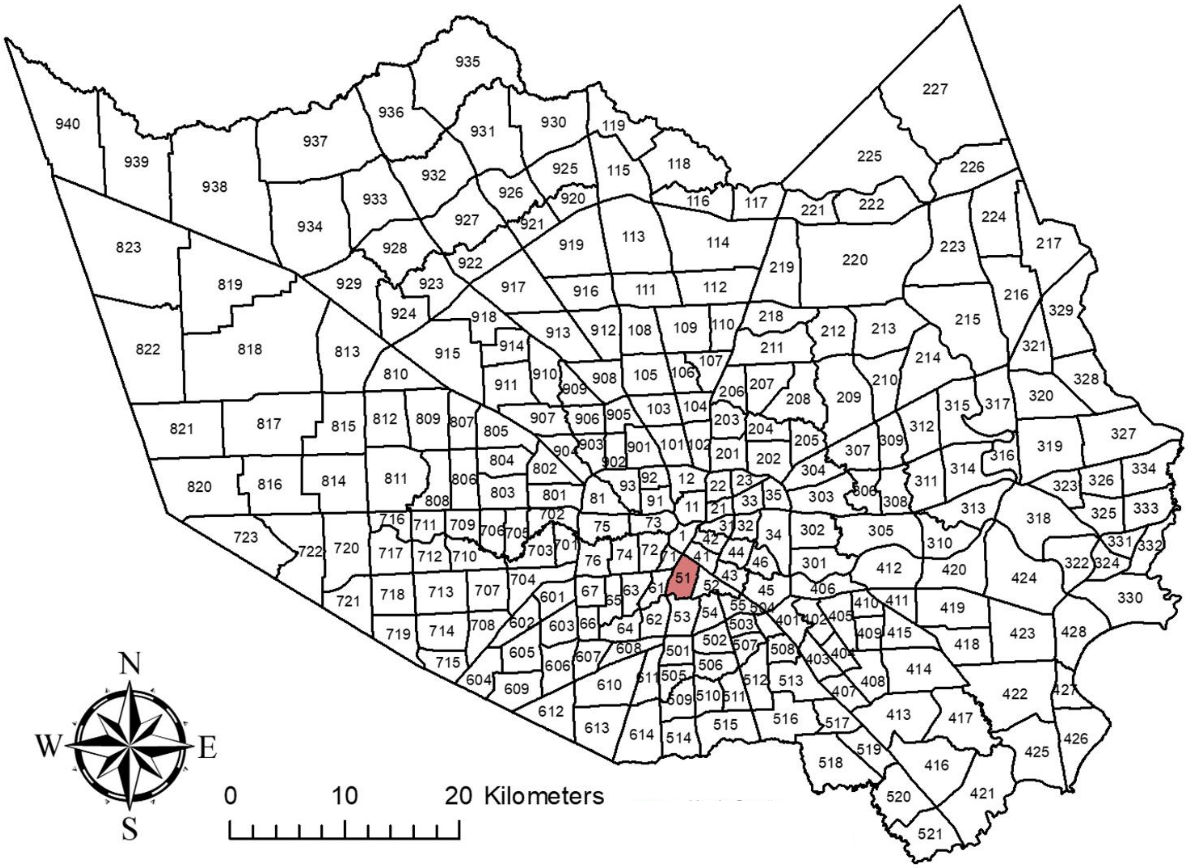

Powered by Esri
